# Review of Published ECTAS Data Set From 2012/2013 to 2021

**DOI:** 10.1192/bjo.2024.111

**Published:** 2024-08-01

**Authors:** Vellingiri Raja Badrakalimuthu

**Affiliations:** Surrey & Borders Partnership NHS Foundation Trust, Guildford, United Kingdom

## Abstract

**Aims:**

Electroconvulsive Therapy Accreditation Service (ECTAS) publishes minimal data set collected from ECT services subscribing to ECTAS accreditation. The aim of this study is to review minimal data set published by ECTAS towards understanding trends and statistically significant changes between 2013 and 2021.

**Methods:**

ECTAS minimal data set has been published for years 2012/13, 2014/15, 2016/17 and 2021. Number of courses, age, gender, diagnoses, legal status, number of treatments, and score on Clinical Global Impression Scale (CGI) for acute treatments has been analysed for trends and statistical differences.

**Results:**

ECTAS data set was not published for the years 2018, 2019 and 2020. In terms of number of courses of treatment per year, 2014/15 was highest with 2148 courses and lowest was in 2016/2017 with 1821 courses. Average number of treatments was 1995 and there was no statistical difference between the years. There was no statistical difference with mean age (61), gender (female 66%), and diagnosis (depression 87.5%). In terms of diagnosis though, there is better documentation of diagnosis in 2021, rather than broad categories such as catatonia used previously, and this has led to schizophrenia as diagnosis in 4% and mixed affective disorder in 5%.

There has been a gradual but not statistically significant trend to increase in treatments per course from 9.3 in 2012/13 to 10.1 in 2021. There is significant increase in number of patients detained at the start of treatment from 42% in 2012/13 to 57% in 2021. Percentage of people in moderate to amongst severely ill categories on CGI at the start has remained the same through the years (mean 96%). CGI at the end of treatment minimal improved to much improved has similarly remained through the year (mean 91%).

The 2021 data set includes subjective memory score with categories showing increases after ECT were 2 (“occasional increased lapses of memory”) and the yet milder category of 1.

**Conclusion:**

Between the published data sets, there is no statistical difference apart from number of patients commencing ECT under the Mental Health Act. This may reflect increasingly better practice in assessing mental capacity, with a greater tendency to appropriate application of Mental Health Act legal framework ensuring legal safeguards for the patient such as right to appeal and statutory access to second opinion.

